# Lack of Efficacy of Combined Carbohydrate Antigen Markers for Lung Cancer Diagnosis

**DOI:** 10.1155/2020/4716793

**Published:** 2020-12-10

**Authors:** Zhineng Wen, Ying Huang, Zhougui Ling, Jifei Chen, Xiaomou Wei, Rui Su, Zhenming Tang, Zhongwei Wen, Youping Deng, Zhuojun Hu

**Affiliations:** ^1^Department of Pulmonary and Critical Care Medicine, The Fourth Affiliated Hospital of Guangxi Medical University, No. 1, Liushi Road, Liuzhou 545000, China; ^2^Clinical Laboratory, The Fourth Affiliated Hospital of Guangxi Medical University, No. 1, Liushi Road, Liuzhou 545000, China; ^3^Department of Quantitative Health Sciences, University of Hawaii John A. Burns School of Medicine, Honolulu, HI 96813, USA

## Abstract

**Background:**

Lung cancer (LC) is top-ranked in cancer incidence and is the leading cause of cancer death globally. Combining serum biomarkers can improve the accuracy of LC diagnosis. The identification of the best potential combination of traditional tumor markers is essential for LC diagnosis. *Patients and Methods*. Blood samples were collected from 132 LC cases and 118 benign lung disease (BLD) controls. The expression levels of ten serum tumor markers (CYFR21, CEA, NSE, SCC, CA15-3, CA 19-9, CA 125, CA50, CA242, and CA724) were assayed, and that the expression in the levels of tumor markers were evaluated, isolated, and combined in different patients. The performance of the biomarkers was analyzed by receiver operating characteristic (ROC) analyses, and the difference between combinations of biomarkers was compared by Chi-square (*χ*^2^) tests.

**Results:**

As single markers, CYFR21 and CEA showed good diagnostic efficacy for nonsmall cell lung cancer (NSCLC) patients, while NSE and CEA were the most sensitive in the diagnosis of small cell lung cancer (SCLC). The area under the curve (AUC) value was 0.854 for the panel of four biomarkers (CYFR21, CEA, NSE, and SCC), 0.875 for the panel of six biomarkers (CYFR21, CEA, NSE, SCC, CA125, and CA15-3), and 0.884 for the panel of ten markers (CYFR21, CEA, NSE, SCC, CA125, CA15-3, CA19-9, CA50, CA242, and CA724). With a higher sensitivity and negative predictive value (NPV), the diagnostic accuracy of the three panels was better than that of any single biomarker, but there were no statistically significant differences among them (all *P* values > 0.05). However, the panel of six carbohydrate antigen (CA) biomarkers (CA125, CA15-3, CA19-9, CA50, CA242, and CA724) showed a lower diagnostic value (AUC: 0.776, sensitivity: 59.8%, specificity: 73.0%, and NPV: 60.4%) than the three panels (*P* value < 0.05). The performance was similar even when analyzed individually by LC subtypes.

**Conclusion:**

The biomarkers isolated are elevated for different types of lung cancer, and the panel of CYFR21, CEA, NSE, and SCC seems to be a promising serum biomarker for the diagnosis of lung cancer, while the combination with carbohydrate antigen markers does not improve the diagnostic efficacy.

## 1. Introduction

In the United States, lung cancer (LC) has the second-highest incidence rate of new cases and the highest cancer-related death rate in both men and women [1] (Siegel, Miller et al. 2019). Globally, low- and middle-income countries now estimate more than 50% of mortality every year [2] (Torre, Siegel et al. 2016). There are three main histological subtypes: squamous cell carcinomas (SCC), adenocarcinomas (AdC), and small cell carcinoma [3] (Lortet-Tieulent, Soerjomataram et al. 2014) [4, 5] (Adjei 2019, Feng, Zong et al. 2019). Early identification and surgical resection are regarded as the mainstay to reduce the mortality of lung cancer patients [6] (Satoh, Hoshi et al. 2007). Currently, the initial diagnostic approaches for lung cancer include clinical presentations, chest X-ray, computed tomography (CT) scans, blood biomarkers, and biopsy. Since many of the symptoms of lung cancer are common but nonspecific, in primary care practice, it is very difficult to identify lung cancer patients from patients with other benign lung diseases (BLDs) [7] (Weller, Peake et al. 2019). Histopathological examination is the gold standard for the diagnosis of LC, and there are numerous invasive ways to obtain pathological tissue, such as bronchoscopy and biopsy guided by CT scan. However, these invasive approaches are painful, inconvenient, and expensive. Therefore, chest CT and tumor biomarker tests preceding biopsy are essential.

Blood biomarkers are minimally invasive, simple, and relatively inexpensive and have no potential radiological hazards as that of CT examinations. Thus, they could serve as a convenient, complementary method for both the diagnosis and assessment of the possible pathological types of LC [8, 9] (Isaksson, Jonsson et al. 2017, Chen, Huang et al. 2018). Although novel promising biomarkers have been discovered and developed, such as autoantibodies, microRNAs (especially small noncoding RNAs (ncRNAs)), circulating tumor DNA, DNA methylation, complement fragments, blood protein profiling, and RNA airway or nasal signatures, the objective of these new biomarkers is mainly for use in early-stage LC screening [10, 11] (Dou, Zhu et al. 2018, Seijo, Peled et al. 2019). Currently, traditional tumor-associated antigen (TAA) biomarkers, such as cytokeratin 19 fragment antigen 21-1 (CYFRA21-1), neuron-specific enolase (NSE), carcinoembryonic antigen (CEA), squamous cell carcinoma antigen (SCC), carbohydrate antigen (CA) 125, CA15-3, CA19-9, and CA72-4, remain widely used as reference diagnoses for lung cancer [9, 10] (Isaksson, Jonsson et al. 2017, Seijo, Peled et al. 2019). Single markers have limited sensitivity and specificity, and combinations of tumor markers (TMs) can improve the diagnostic accuracy [12–15] (Yoon, Kwon et al. 2016, Li, Zhang et al. 2017, Liu, Teng et al. 2017, Fang, Zhu et al. 2018), but few studies have compared different panels of TAAs, and some of these biomarkers may be increased in patients with BLD. Therefore, the aim of this study was to identify the best potential combinations of these traditional tumor markers in distinguishing lung cancer patients from BLD controls.

## 2. Materials and Methods

### 2.1. Patients

This was a retrospective study approved by the Ethics Committee of the Fourth Affiliated Hospital of Guangxi Medical University (number KY2019208). We systematically reviewed all patients who had been hospitalized at the Department of Pulmonary and Critical Care Medicine of the hospital between January 2017 and May 2019. Written informed consent was provided by each participant. The patients' clinicopathological information, including age, sex, smoking history, and lung cancer histology, was obtained from electronic medical records. A “never smoker” was defined as a person who had smoked less than 100 cigarettes during his/her lifetime. LC was defined based on CT scans and verified by histopathology according to the World Health Organization Classification of Tumors [16] (Travis, Brambilla et al. 2015). The diagnosis of BLD was established by clinical data and CT scans. Complete and detailed case information was available for all cases.

### 2.2. Tumor Marker Measurement

Fasting blood samples were collected the morning after patient admission before any therapeutics were administered. Sera were separated after blood collection, and the tests were completed immediately to avoid attenuation. The serum concentrations of TAAs were quantitated by an electrochemiluminescence immunoassay at the Clinical Laboratory of the Fourth Affiliated Hospital of Guangxi Medical University. All assays were performed according to the instrument and reagent specifications.

According to the manufacturers, the cutoff values were as follows: CYFR21 < 3.3 ng/mL, CEA < 3.4 ng/mL, NSE < 16.3 ng/mL, SCC < 2.5 ng/mL, CA125 < 36 U/mL, CA153 < 25 U/mL, CA199 < 27 U/mL, CA50 < 25 U/mL, CA242 < 10 U/mL, and CA724 < 6 U/mL. The laboratory technicians were blinded to the patients' identity, but the results were unblinded and analyzed by our investigators.

### 2.3. Statistical Analysis

The data are described as means with standard deviations (SDs) for the continuous variables and frequencies with percentages for the categorical variables. The differences in the serum levels between LC patients and BLD controls were compared using a two-sample Mann–Whitney *U* test. The sensitivity, specificity, negative predictive value (NPV), and positive predictive value (PPV) were calculated to evaluate the performance of combination panels and the different biomarkers. The area under the curve (AUC) and standard error (SE) for the respective receiver operating characteristic (ROC) curves were calculated. An AUC greater than 0.9 implied excellent diagnostic efficacy, whereas an AUC between 0.7 and 0.9 implied good diagnostic efficacy, and an AUC between 0.5 and 0.7 implied poor diagnostic efficacy. Finally, an AUC of no more than 0.5 implied the lack of a diagnostic value for the marker [17] (Yang, Zhang et al. 2018). The ROC curves were constructed by calculating the sensitivity/specificity of the test for a succession of deviations from the original cut-offs, with the same deviation for each antigen in the panel. Chi-square (*χ*^2^) tests were used to compare a significant difference between two panels of biomarkers. All analyses were conducted using SPSS 20.0 (SPSS Inc., Chicago, IL, USA), MedCalc 18.2, and GraphPad Prism 5.0 software (GraphPad Software Inc., San Diego, CA, USA). A 2-sided *P* value < 0.05 was considered statistically significant.

## 3. Results

### 3.1. Patients' Characteristics

A total of 132 patients with pathologically confirmed lung cancer (including 102 with nonsmall cell lung cancer (NSCLC) and 30 with small cell lung cancer (SCLC)), and 118 patients with benign lung diseases were included in the study. There were more LC patients in the late stage (III-IV) (87.9%) than in the early stage (I-II) (12.1%), and the LC subtypes included adenocarcinoma (55.2%), squamous-cell carcinoma (22.0%), SCLC (22.0%), and large cell lung carcinoma (0.8%). The etiologic diagnoses of the BLD group included bronchitis, community-acquired pneumonia (CAP), chronic obstructive pulmonary disease (COPD), cough variant asthma (CVA), obstructive sleep apnea syndrome (OSAS), bronchiectasis, parapneumonic effusion, and pulmonary tuberculosis. Age, sex, and history of smoking did not differ significantly between the groups (*P* > 0.05).  Table 1 summarizes the patients' characteristics.

### 3.2. The Significance of Single Markers for LC Screening

**Figure 1:**
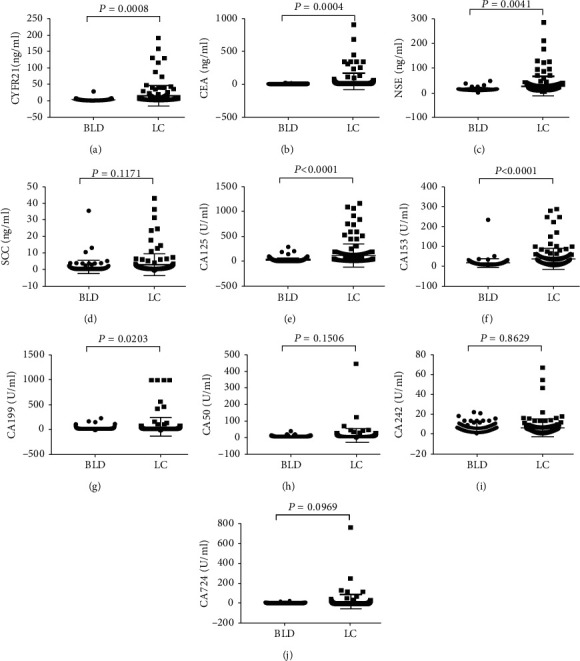
Concentrations of each biomarker between lung cancer (LC) cases and benign lung diseases (BLD) controls. The bold horizontal lines in the box plots are medians, and the lower and upper limits of the boxes are 25th and 75th percentiles of values, respectively. The *P* values were obtained from Mann–Whitney *U* test.

**Figure 2:**
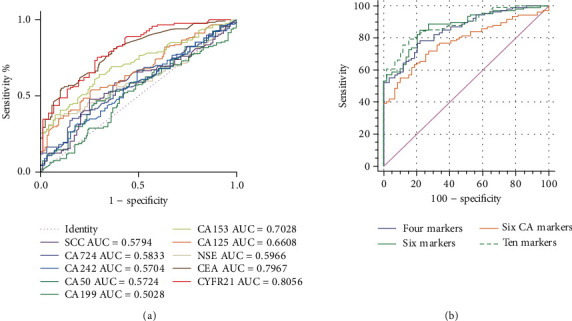
ROC curves to assess the value of single marker and combined markers in lung cancer patients versus benign lung disease controls. (a) ROC of single marker; (b) ROC of combined markers.

To determine the reactivity of a single marker, ten TAAs (CYFR21, CEA, NSE, SCC, CA15-3, CA 19-9, CA 125, CA50, CA242, and CA724) were measured from 132 LC patients and 118 BLD controls. The results showed that the serum concentrations of six biomarkers were significantly higher in LC patients than in BLD controls: CYFR21 (*P* = 0.008), CEA (*P* = 0.004), NSE (*P* = 0.0041), CA125 (*P* < 0.0001), CA15-3 (*P* < 0.0001), and CA19-9 (*P* = 0.0203). The serum concentrations of the remaining four TAAs were similar in the LC patients to those in the BLD controls: SCC (*P* = 0.117), CA50 (*P* = 0.151), CA242 (*P* = 0.863), and CA724 (*P* = 0.097) (Figure 1). Among the ten serum biomarkers, CYFR21 displayed the highest AUC (0.806, 95% CI: 0.743-0.859) with a sensitivity of 73.5%, followed by CEA (AUC = 0.797, 95% CI: 0.741–0.845; sensitivity: 65.4%) and CA15-3 (AUC = 0.703, 95% CI: 0.642–0.759; sensitivity: 37.1%). The other markers demonstrated poor diagnostic efficacy for lung cancer screening, with low AUC values (<0.7) and similarly low sensitivities. The ROC curve and screening value of each marker for identifying lung cancer patients and BLD patients can be found in Figure 2(a) and Table 2.

### 3.3. The Value of the Combined Detection of Markers for the Detection of Lung Cancer

To assess the diagnostic value of the combination of TAAs in distinguishing LC from BLD, we evaluated different combinations of TAA markers. We combined biomarkers into four different panels: panel 1 containing four biomarkers: CYFR21, CEA, NSE, and SCC; panel 2 containing six biomarkers: CYFR21, CEA, NSE, SCC, CA125, and CA15-3; panel 3 containing only the six CA markers: CA125, CA15-3, CA19-9, CA50, CA242, and CA724; and panel 4 containing all ten biomarkers: CYFR21, CEA, NSE, SCC, CA125, CA15-3, CA19-9, CA50, CA242, and CA724. The AUC, sensitivity, specificity, PPV, and NPV for the tumor marker combinations were as follows: panel 1 (0.854, 89.4%, 61.9%, 72.4%, and 83.9%), panel 2 (0.875, 95.5%, 57.6%, 71.6%, and 91.9%), panel 3 (0.776, 59.8%, 73.0%, 72.5%, and 60.4%), and panel 4 (0.884, 95.5%, 52.5%, 69.2%, and 91.2%) (Figure 2(b), Table 2). Except for panel 3, which contained six CA biomarkers, the performance of three panels of biomarkers was better than that of any single marker (Table 2). However, panel 3 has good diagnostic efficacy and is shown to be less than two individual biomarkers (CYFR21 and CEA) and superior to the rest of the unique biomarkers (8 biomarkers).

### 3.4. The Best Marker Combinations for Lung Cancer Screening

To further investigate the value of the four combinations of TAA markers and to identify the best potential panel of biomarkers for LC diagnosis, we performed pairwise comparisons. Without obvious differences in AUC values, the diagnostic value of panel 1 was not inferior to that of panel 2 (all *P* values > 0.05); there was also no significant difference between panels 1 and 2 compared with panel 4 (all *P* values > 0.05) (Table 3). However, panel 3, consisting of only CA biomarkers, showed a lower diagnostic accuracy than any of the other three panels; its AUC, sensitivity, and NPV were significantly lower than those of the three panels. Interestingly, adding two of the CA biomarkers (CA125 and CA15-3) to panel 1 to create panel 2 did not improve the diagnostic efficacy.

### 3.5. Diagnostic Efficacy of the Biomarkers for Lung Cancer Subtypes

**Figure 3:**
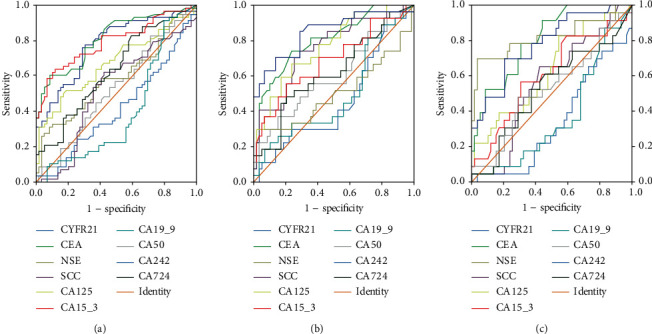
ROC curves to assess the value of single marker in lung cancer subtypes. (a) Adenocarcinoma; (b) squamous-cell carcinoma; (c) small cell lung cancer.

**Figure 4:**
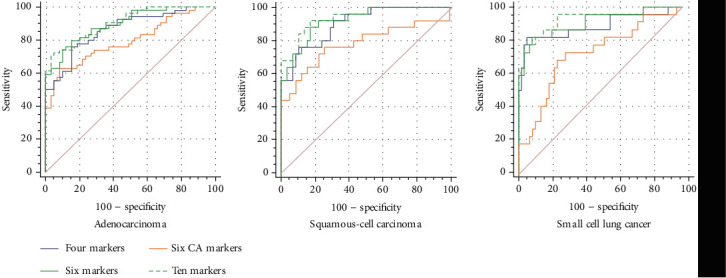
ROC curves to assess the value of combined markers in lung cancer subtypes.

To evaluate the diagnostic value of the biomarkers for different subtypes of LC, we performed further histological classification of the patients with lung cancer. For the performance of single markers, CEA (AUC: 0.812; sensitivity: 63.9%) was the most related to lung adenocarcinoma (Figure 3(a), Table 4); CYFR21 (AUC: 0.847; sensitivity: 84.6%) and CEA (AUC: 0.804; sensitivity: 70.0%) were the best suited for squamous-cell carcinoma (Figure 3(b), Table 5); and NSE (AUC: 0.819; sensitivity: 69.0%) and CEA (AUC: 0.808; sensitivity: 60.7%) were the most related to SCLC (Figure 3(c), Table 6). For the performance of the combined markers, without obvious differences in AUC and sensitivity values, there was no significant difference among panels 1, 2, and 4, and all of them showed a higher diagnostic accuracy than panel 3 (Figure 4, Tables 3–5).

## 4. Discussion

Tumor biomarkers are mainly used for potential diagnosis and for monitoring the efficacy of therapy in lung cancer patients. In this study, we evaluated the detection value of single or combined serum biomarkers for individuals with potential LC. Our results confirmed that CYFRA21-1 and CEA were the most sensitive as a single marker in the diagnosis of NSCLC, while NSE was the best suited, followed by CEA, for the diagnosis of SCLC, which is in accordance with previous reports [14, 18, 19] (Molina, Agusti et al. 1994, Vinolas, Molina et al. 1998, Liu, Teng et al. 2017). We also found that other individual biomarkers yielded low efficacy for diagnosing LC, especially some CA markers. Even in combination, the CA markers maintained poor diagnostic efficacy with low AUC, sensitivity, and NPV. In contrast, some combinations of multiple markers were shown to improve the diagnostic accuracy over their individual component biomarkers. Our data demonstrated that improved diagnostic accuracy can be achieved with panels combining biomarkers. In particular, panel 1, consisting of 4 TAAs (CYFR21, CEA, NSE, and SCC), panel 2, consisting of 6 TAAs (CYFR21, CEA, NSE, SCC, CA125, and CA15-3), and panel 4, consisting of 10 TAAs (CYFR21, CEA, NSE, SCC, CA125, CA15-3, CA19-9, CA50, CA242, and CA724), showed improved diagnostic efficacy in different types of lung cancer patients. Even when analyzed separately by LC subtypes, their performance was similar. The lack of a difference between their AUCs could be accounted for by assuming that the addition of more markers could not improve the detection rate of LC. However, we did not evaluate the efficacy of combined markers specifically in early-stage patients because of the small number of these patients in this study.

It is well known that different lung cancers have the same antigens, and a single type of cancer will elevate different biomarkers. The value of the detection of single markers for identifying patients with lung cancer is low; therefore, many others have identified combinations of TAA biomarkers that are useful for detecting LC. Most recently, Qu et al. [20] (Qu, Zhang et al. 2019) showed that the combination of thioredoxin (Trx), CYFRA21-1, and SCC biomarkers can improve both specificity and sensitivity to diagnose lung cancer patients from healthy subjects. Jiang et al. [21] (Jiang, Wang et al. 2018) investigated a combination of CEA, CYFRA21, NSE, and thymidine kinase 1 (TK1) for lung cancer diagnosis; the results showed that the diagnostic value of TK1 combined with CEA, CYFRA21-1, and NSE was significantly higher than that of each biomarker alone. In addition, TK1 combined with CEA, CYFRA21-1, or NSE could improve the diagnosis of squamous cell carcinoma, adenocarcinoma, or small cell lung cancer, respectively. Mazzone et al. [22] (Mazzone, Wang et al. 2018) also validated a panel of biomarkers, including hepatocyte growth factor (HGF), CEA, CYFRA21-1, CA125, and New York esophageal cancer-1 antibody (NY-ESO-1), together with clinical variables (including age, sex, COPD status, and smoking history). They reported an increase in AUC from 0.81 to 0.86 for the biomarkers combined with the clinical variables, with a specificity of 88% and sensitivity of 67%. Yoon et al. [15] (Yoon, Kwon et al. 2016) discovered a combination of six biomarkers (HE4, CEA, RANTES, ApoA2, TTR, and sVCAM-1) with a sensitivity of 93.33% and specificity of 92.00% in the diagnosis of lung cancer. Liu et al. [14] (Liu, Teng et al. 2017) found that the combination of six tumor markers (CEA, CYFRA21-1, SCC, NSE, ProGRP, and CA125) could discriminate the histological types of lung cancer between SCLC and NSCLC. Korkmaz et al. [23] (Korkmaz, Koksal et al. 2018) suggested a panel of three serum tumor biomarkers, CYFRA21-1, HE4, and progastrin-releasing peptide (ProGRP) that might contribute to discriminating lung cancer from BLD. They reported an increase in the diagnostic value (AUC = 0.899) for CYFRA21-1 combined with HE4 for discriminating LC from BLD, while ProGRP alone had the highest diagnostic value (AUC = 0.875) for discriminating SCLC from NSCLC. Fang et al. [13] (Fang, Zhu et al. 2018) found that a panel of PRL, CEA, and CYFRA21 biomarkers was more effective than the individual biomarkers alone, with a relatively high sensitivity and specificity for the diagnosis of NSCLC. Consistent with these observations, we found that some panels of combined TAA biomarkers yielded a better, more optimal diagnostic efficacy for cancer patients than the individual biomarkers alone. We found that the panel consisting of 4 TAAs (CYFR21, CEA, NSE, and SCC) showed good diagnostic efficacy for lung cancer screening in BLD, with an AUC of 0.854 and a sensitivity of 89.4%. The diagnostic value of the four-TAA combination was not inferior to that of the panel of 6 TAAs (CYFR21, CEA, NSE, SCC, CA125, and CA15-3) or 10 TAAs (CYFR21, CEA, NSE, SCC, CA125, CA15-3, CA19-9, CA50, CA242, and CA724) because there were no statistically significant differences among them (all *P* values > 0.05). Even when analyzed individually by LC subtypes, the performance of these combinations was also similar. This means that the addition of more markers did not improve the detection rate of LC patients.

However, not all combinations of biomarkers improve diagnostic efficacy. Mauro et al. [24] (Mauro, Passerini et al. 2019) found that using two markers (ProGRP and NSE) did not increase the accuracy over either marker individually for small cell lung cancer diagnosis. Yang et al. [17] (Yang, Zhang et al. 2018) also showed that a two-marker combination is more suitable than a multimarker combination for the serological screening of tumors. In the present study, we found that a panel of six CA biomarkers (CA125+ CA15-3+ CA19-9+ CA50+ CA242+ CA724) did not yield better diagnostic efficacy for lung cancer patient screening, whether overall or for LC subtypes. The diagnostic accuracy of the combination including CA markers was not only inferior to that of the other three panels but was also not superior to some of the single biomarkers, such as CYFRA21 or CEA. It is probable that the reason is linked to the similar serum concentrations of three of the six CA markers (CA50+ CA242+ CA724) between LC patients and BLD controls, and others demonstrate poor diagnostic efficacy for lung cancer screening. The serum CA levels not only increase in lung cancer and other types of cancers but also in nonneoplasm diseases and healthy individuals [25–29] (Glasgow, Pacheco-Rodriguez et al. 2018, Dou, Sun et al. 2019, Shan, Tian et al. 2019, Zeng, Li et al. 2019, Zhang, Zhang et al. 2019). However, even adding CA125 and CA15-3 into the panel with 4 markers (CEA + CY211 + NSE + SCC) could not improve the diagnostic efficacy of LC and BLD identification. These results indicate that a single biomarker or a panel that includes CA biomarkers is not a good choice for this purpose.

In conclusion, CYFR21 and CEA were elevated in patients with LC and could potentially be used as effective single serum biomarkers for the diagnosis of NSCLC, while NSE and CEA were the most sensitive for the diagnosis of SCLC. As a less expensive test to distinguish between LC and BLD in general practice clinics, the combination of four serum biomarkers (CYFR21, CEA, NSE, and SCC) seems more promising for the diagnosis of lung cancer and should be used in common practice. However, combinations including CA biomarkers may not be appropriate for diagnosing lung cancer. Our research also has some limitations. Due to the lack of samples, the efficacy of combined markers specifically in early-stage patients needs to be improved. We will collect more samples of early-stage patients to verify our findings.

## Figures and Tables

**Table 1:** Clinical characteristics of the LC patients and BLD controls.

Parameters	LC (*n* = 132)	BLD (*n* = 118)	*P* value
Age (year)			
Range	40-82	35-87	
Mean (SD)	61.4 (9.9)	57.9 (10.6)	0.164
Gender			
Male	106	84	0.092
Female	26	34	0.092
Smoking, *n* (%)			
Ever/current	89 (67.4)	72 (61.0)	0.291
Never	43 (32.6)	46 (39.0)	0.291
Cancer stage, *n* (%)		Diseases (*n*)	
I	6 (4.5)	Bronchitis (26)	
II	10 (7.6)	CAP (55)	
III	47 (35.6)	COPD (8)	
IV	69 (52.3)	Bronchiectasis (12)	
Cancer subtype, *n* (%)		Pulmonary tuberculosis (6)	
Adenocarcinoma	73 (55.2)	Parapneumonic effusion (4)	
Squamous-cell carcinoma	29 (22.0)	OSAS (4)	
Large cell lung carcinoma	1 (0.8)	CVA (3)	
SCLC	29 (22.0)		

SD: standard deviation; LC: lung cancer; SCLC: small cell lung cancer; BLD: benign lung diseases; CAP: community-acquired pneumonia; COPD: chronic obstructive pulmonary disease; CVA: cough variable asthma; OSAHS: obstructive sleep apnea syndrome.

**Table 2:** Performances of biomarkers in lung cancer diagnosis.

Biomarkers	Sensitivity	Specificity	PPV	NPV	AUC (95% CI)
CYFR21	0.735	0.732	0.824	0.619	0.806 (0.743-0.859)
CEA	0.654	0.827	0.817	0.669	0.797 (0.741-0.845)
NSE	0.444	0.757	0.757	0.444	0.597 (0.525-0.665)
SCC	0.192	0.848	0.862	0.426	0.579 (0.511-0.645)
CA125	0.400	0.879	0.833	0.493	0.661 (0.598-0.720)
CA15-3	0.371	0.951	0.920	0.500	0.703 (0.642-0.759)
CA199	0.190	0.915	0.786	0.478	0.503 (0.439-0.567)
CA50	0.063	0.988	0.889	0.411	0.572 (0.494-0.651)
CA242	0.095	0.871	0.522	0.394	0.570 (0.492-0.649)
CA724	0.157	0.941	0.800	0.428	0.583 (0.507-0.660)
The panel 1	0.894	0.619	0.724	0.839	0.854 (0.791-0.904)
The panel 2	0.955	0.576	0.716	0.919	0.875 (0.815-0.921)
The panel 3	0.598	0.730	0.725	0.604	0.776 (0.705-0.837)
The panel 4	0.955	0.525	0.692	0.912	0.884 (0.825-0.928)

PPV: positive predictive value; NPV: negative predictive value; Pane l = CYFR21, CEA, NSE, and SCC; panel 2 = CYFR21, CEA, NSE, SCC, CA125, and CA15-3; panel 3 = CA125, CA15-3, CA19-9, CA50, CA242, and CA724; panel 4 = CYFR21, CEA, NSE, SCC, CA125, CA15-3, CA19-9, CA50, CA242, and CA724.

**Table 3:** Compare the four panels of biomarkers in lung cancer diagnosis.

Panels	Sensitivity (*P* value)	Specificity (*P* value)	PPV (*P* value)	NPV (*P* value)	AUC (*P* value)
Panel 1 vs. panel 2	0.063	0.507	0.870	0.126	0.194 (*z* = 1.300)
Panel 1 vs. panel 3	<0.0001	0.073	0.988	<0.0001	0.039 (*z* = 2.06)
Panel 1 vs. panel 4	0.063	0.148	0.519	0.180	0.10 (*z* = 1.66)
Panel 2 vs. panel 3	<0.001	0.015	0.871	<0.0001	<0.0001 (*z* = 3.44)
Panel 2 vs. panel 4	1.000	0.432	0.625	0.878	0.387 (*z* = 0.86)
Panel 3 vs. panel 4	<0.001	0.001	0.557	<0.0001	<0.0001 (*z* = 4.25)

Panel = CYFR21, CEA, NSE, and SCC; panel 2 = CYFR21, CEA, NSE, SCC, CA125, and CA15-3; panel 3 = CA125, CA15-3, CA19-9, CA50, CA242, and CA724; panel 4 = CYFR21, CEA, NSE, SCC, CA125, CA15-3, CA19-9, CA50, CA242, and CA724.

**Table 4:** Performances of biomarkers in lung adenocarcinoma diagnosis.

Biomarkers	Sensitivity	Specificity	PPV	NPV	AUC (95% CI)
CYFR21	0.766	0.732	0.721	0.776	0.777 (0.693-0.862)
CEA	0.639	0.827	0.667	0.787	0.812 (0.734-0.890)
NSE	0.409	0.757	0.600	0.589	0.589 (0.485-0.694)
SCC	0.123	0.848	0.643	0.549	0.523 (0.415-0.630)
CA125	0.414	0.879	0.744	0.640	0.674 (0.575-0.773)
CA15-3	0.471	0.951	0.892	0.678	0.793 (0.710-0.876)
CA199	0.121	0.915	0.727	0.356	0.399 (0.293-0.505)
CA50	0.042	0.988	0.750	0.547	0.525 (0.420-0.630)
CA242	0.129	0.871	0.450	0.548	0.416 (0.312-0.519)
CA724	0.192	0.941	0.700	0.627	0.640 (0.541-0.740)
The panel 1	0.849	0.619	0.579	0.869	0.864 (0.787-0.922)
The panel 2	0.867	0.576	0.561	0.883	0.897 (0.826-0.946)
The panel 3	0.644	0.730	0.595	0.768	0.794 (0.708-0.864)
The panel 4	0.945	0.525	0.552	0.939	0.904 (0.834-0.951)

**Table 5:** Diagnostic efficacy of biomarkers with squamous-cell carcinoma.

Biomarkers	Sensitivity	Specificity	PPV	NPV	AUC (95% CI)
CYFR21	0.846	0.732	0.537	0.929	0.847 (0.749-0.945)
CEA	0.700	0.827	0.500	0.914	0.804 (0.702-0.905)
NSE	0.321	0.757	0.333	0.747	0.501 (0.350-0.651)
SCC	0.517	0.848	0.517	0.848	0.758 (0.646-0.870)
CA125	0.464	0.879	0.565	0.830	0.767 (0.661-0.874)
CA15-3	0.321	0.951	0.692	0.804	0.687 (0.559-0.816)
CA199	0.310	0.915	0.750	0.615	0.493 (0.357-0.630)
CA50	0.138	0.988	0.800	0.766	0.599 (0.463-0.735)
CA242	0.172	0.871	0.313	0.755	0.456 (0.322-0.590)
CA724	0.172	0.941	0.455	0.800	0.622 (0.490-0.753)
The panel 1	0.966	0.619	0.364	0.986	0.903 (0.819-0.957)
The panel 2	0.966	0.576	0.359	0.986	0.923 (0.843-0.970)
The panel 3	0.724	0.730	0.396	0.915	0.782 (0.678-0.864)
The panel 4	0.966	0.525	0.333	0.984	0.939 (0.865-0.980)

**Table 6:** Diagnostic efficacy of biomarkers with SCLC.

Biomarkers	Sensitivity	Specificity	PPV	NPV	AUC (95% CI)
CYFR21	0.586	0.732	0.472	0.813	0.783 (0.672-0.894)
CEA	0.607	0.827	0.459	0.897	0.808 (0.711-0.905)
NSE	0.690	0.757	0.526	0.862	0.819 (0.696-0.943)
SCC	0.074	0.848	0.125	0.757	0.557 (0.425-0.690)
CA125	0.269	0.879	0.412	0.793	0.633 (0.498-0.768)
CA15-3	0.160	0.951	0.500	0.788	0.607 (0.466-0.748)
CA199	0.214	0.915	0.667	0.593	0.367 (0.238-0.497)
CA50	0.071	0.988	0.667	0.759	0.531 (0.387-0.674)
CA242	0.077	0.871	0.154	0.755	0.340 (0.213-0.467)
CA724	0.038	0.941	0.143	0.793	0.529 (0.385-0.673)
The panel 1	0.931	0.619	0.355	0.972	0.884 (0.794-0.945)
The panel 2	0.931	0.576	0.659	0.882	0.908 (0.822-0.961)
The panel 3	0.483	0.730	0.304	0.851	0.720 (0.609-0.814)
The panel 4	0.931	0.525	0.325	0.969	0.928 (0.848-0.973)

## Data Availability

The data used to support the findings of this study are available from the corresponding author upon request.
